# Evaluation of Diet-Related Infographics on Pinterest for Use of Behavior Change Theories: A Content Analysis

**DOI:** 10.2196/mhealth.6367

**Published:** 2016-12-08

**Authors:** Jessica L Wilkinson, Kate Strickling, Hannah E Payne, Kayla C Jensen, Joshua H West

**Affiliations:** ^1^ Computational Health Science Research Group Department of Health Science Brigham Young University Provo, UT United States

**Keywords:** behavioral health, content analysis, nutrition, social media, Internet, healthy eating, theory

## Abstract

**Background:**

There is increasing interest in Pinterest as a method of disseminating health information. However, it is unclear whether the health information promoted on Pinterest is evidence-based or incorporates behavior change theory.

**Objectives:**

The objective of the study was to determine the presence of health behavior theory (HBT) constructs in pins found on Pinterest and assess the relationship between various pin characteristics and the likelihood of inclusion of HBT.

**Methods:**

A content analysis was conducted on pins collected from Pinterest identified with the search terms “nutrition infographic” and “healthy eating infographic.” The coding rubric included HBT constructs, pin characteristics, and visual communication tools. Each HBT construct was coded as present or not present (yes=1, no=0). A total theory score was calculated by summing the values for each of the 9 constructs (range 0-9). Adjusted regression analysis was used to identify factors associated with the inclusion of health behavior change theory in pins (*P*<.05).

**Results:**

The mean total theory score was 2.03 (SD 1.2). Perceived benefits were present most often (170/236, 72%), followed by behavioral capability (123/238, 51.7%) and perceived severity (79/236, 33.5%). The construct that appeared the least was self-regulation/self-control (2/237, 0.8%). Pin characteristics associated with the inclusion of HBT included a large amount of text (*P*=.01), photographs of real people (*P*=.001), cartoon pictures of food (*P*=.01), and the presence of references (*P*=.001). The number of repins (*P*=.04), likes (*P*=.01), and comments (*P*=.01) were positively associated with the inclusion of HBT.

**Conclusions:**

These findings suggest that current Pinterest infographics targeting healthy eating contain few HBT elements. Health professionals and organizations should create and disseminate infographics that contain more elements of HBT to better influence healthy eating behavior. This may be accomplished by creating pins that use both text and images of people and food in order to portray elements of HBT and convey nutritional information.

## Introduction

Chronic disease is a major public health concern. In the United States, heart disease, cancer, and stroke cause over 50% of all deaths [1]; globally, this figure is 60% [2]. While the etiology of obesity and related chronic diseases is multifactorial, poor diet is one of the most prominent risk factors [3]. The quality of the average American diet has decreased over the past several decades as sugar, salt, and trans fat consumption increased continually, resulting in an increased average caloric intake [4-6]. The Centers for Disease Control and Prevention reports that Americans are consuming a median of 1.8 servings of vegetables and 1.1 servings of fruit per day compared to the 2 to 3 servings of vegetables and 2 servings of fruit recommended [7,8]. Promoting dietary modifications is one of the most important chronic disease prevention strategies [9]. The benefits of improved diet are not limited to disease prevention but also include positive health outcomes such as increased life expectancy, stable body weight, and improved mental health [10-12].

In an attempt to alter these unhealthy eating trends, health professionals are increasingly using the Internet to improve dietary behaviors in populations [13,14]. The Internet, including social media, has become a means of communicating health information [15]; in 2008, more than half of adult patients reported searching online for health information, and searching for health information was the most popular online activity for adults after email and general searches [16]. Pinterest, a social media platform that allows users to share content through photos and pinboards, is one site that has become a repository of health information, both formal and informal. A total of 40% of daily pinners (using Pinterest at least 1 time daily) and 25% of active pinners (using Pinterest at least 1 time monthly) consider Pinterest their “go-to” source for health information; furthermore, 84% of daily pinners reported trying something new once a week or more because of something they saw on Pinterest [17]. Additionally, more people with more diversity are using Pinterest; 67% of users are under age 40 years and 82% are female [17]. Minority group membership is growing with over half of users having joined in the past year [17]. Infographics are a common category of pins shared on Pinterest. Infographics are data visualization tools that aim to communicate information through elements such as graphs and images. Infographics have become increasingly popular in education due to their ability to present complex data in a simple and clear manner and are used by many public health professionals to disseminate health information [18-20]; social media sites like Pinterest may be a useful medium for health educators to share infographics efficiently to large numbers of people.

Pinterest is beginning to impact health educators and the way they share information [21]. Indeed, it is emerging as a tool not just among health professionals [22] but also among many health organizations [23,24]. However, despite the increasing interest in Pinterest as a method of disseminating health information, it is unclear whether the health information promoted on Pinterest is evidence-based or promotes behavior change. This is concerning provided that nutrition and diet information found on the Internet may largely be inaccurate; Hirasawa et al [25] found in a content analysis of online nutrition searches that most advice followed recommended nutrition guidelines only partially. Researchers have conducted content analyses of other health information promoted on social media [26-28] but to date there has been little research about health information found on Pinterest. In one of the few studies of health-related pins on Pinterest, Paige et al [29] reported that chronic obstructive pulmonary disease (COPD) pins incorporated significant levels of verbal persuasion and social modeling and may be useful as a health communication tool for COPD patients [29]. It is uncertain whether diet and nutrition pins are similarly appropriate for distribution by health professionals.

Pinterest may be a useful tool to disseminate information about dietary behaviors, but there is no research about the content of nutrition and diet pins. In particular, it is of interest whether they contain constructs of health behavior theory (HBT), as health promotion materials containing more elements of HBT have been demonstrated to be more effective in changing behavior [30-32]. Specifically, constructs from social cognitive theory [33-35] and health belief model [36-38] have been related to improved dietary practices. Moreover, it may be that approaches to changing dietary practices should incorporate both theories simultaneously [39]. Theory is used to assist a practitioner in organizing information along certain principles believed to change behavior [39]. In the case of Pinterest, then, theory could inform the content of an infographic in order to be most impactful at changing the end user’s behavior. The purpose of this study was to identify a typical sample of infographics and determine the extent to which HBT was integrated. Drawing upon previous research of health technologies we expected HBT to be only marginally represented. Secondarily, we explored the relationship between various pin characteristics and the inclusion of HBT. Whereas there was no previous research to guide this analysis, we provided these data to assist in formulating future research questions and for practitioners wishing to identify the infographics most likely to contain HBT.

## Methods

### Overview

The study protocols met the exemption criteria of the university’s institutional review board. No human subjects were involved in this study and only existing, publicly available data were collected for analysis.

This content analysis evaluated HBT in healthy eating infographic pins selected from Pinterest. Two public health graduate students trained in HBT and nutrition coded the pins to evaluate the extent to which constructs of the health belief model and social cognitive theory, 2 major theories of behavior change, were included. KJ, JLW, and KS are graduate students studying HBT. As it relates to nutritional qualifications, KJ is a registered dietitian nutritionist and was responsible for the nutrition-specific codes in the instrument. JLW has a BS in nutritional science, and KS works professionally as a nutrition coach.

### Pin Selection

The sample was collected from Pinterest in September and October 2015. The study authors created a new Pinterest user account so that no search history would influence the search results. The study sample was identified using the following terms independently entered into the main Pinterest search bar: “nutrition infographic” and “healthy eating infographic.” The first 250 eligible pins that were returned for each term were saved for analysis, resulting in 500 initial pins. Eligible pins included English-language pins that addressed some aspect of nutrition or healthy eating. Examples of this included pins that explained the benefits of vitamins and minerals or provided tips for a healthy diet. Examples of ineligible pins included those addressing physical fitness or the use of food as a beauty product. An Excel spreadsheet (Microsoft Corp) was used to save the URL for each pin and to record the number of repins, likes, and comments. Finally, duplicate pins were deleted resulting in a final sample of 238.

The methodology for identifying pins was adapted from previous eHealth content analysis research [23,40-42]. Whereas previous content analyses of pins identified study samples through boards and then filtered samples by board popularity [29], it was not feasible in the current study because there are too many diet and nutrition boards available on Pinterest and they cannot be sorted by popularity.

### Coding Procedures and Measurements

The researchers coded each pin in the study sample using an HBT-based coding rubric adapted from previous content analysis studies [28,40]. The rubric was adapted to be relevant for pins.

JHW is a senior health communications researcher and trained the other study authors in content analysis research during biweekly sessions over the course of 4 months. First, all authors met to define and reach a common understanding of all the study variables. Second, a coding instrument was developed and pilot tested by jointly coding pins and then resolving any discrepancies in codes. As part of the biweekly training sessions and in response to discrepancies in codes, which were either resolved until agreement was reached or they were removed from the instrument, JHW trained the other study authors to be able to identify the HBT constructs that were measured in this study. Third, the authors revised the instrument to remove coding options for which agreement could not be achieved or that were otherwise determined to be not applicable. Lastly, interrater reliability was established using a subset of the study sample.

The data were coded into an electronic spreadsheet and then exported for analyses. The coding rubric included 4 primary categories: (1) pin characteristics, (2) visual communication tools, (3) health belief model constructs, and (4) social cognitive theory constructs.

Pin characteristics included the URL and the number of repins, likes, and comments for each pin. The variables repins, likes, and comments were not normally distributed and underwent square root transformations for analyses. Pin affiliation/author (business, government, or individual) and the pin category of healthy eating (macronutrient, micronutrient, disease management, portion control/weight management, other) were recorded. Each pin was also coded with respect to whether or not it addressed a particular diet trend, such as paleo (eating only whole unprocessed foods) or avoiding genetically modified foods. Lastly, each pin was assessed to determine whether or not the coder would recommend the pin for use in promoting healthy eating. The coders were uniquely qualified to evaluate this aspect of pins.

The visual communication aspects of each pin were coded. These characteristics included the amount of text in the pin (no text, text light [covering <50% of the pin], or text heavy [covering >50% of the pin]); whether or not there was a person depicted in the pin (yes/no, and if yes, whether or not it was a photo of a real person or a cartoon); whether or not food was depicted in the pin (yes/no, and if yes, whether or not it was a picture of real food or a cartoon); and finally, the dominant colors of the pin (vibrant colors, muted colors, or black and white).

Constructs from social cognitive theory and health belief model were coded. Each HBT construct was coded as present or not present (yes=1, no=0).

### Analysis

To ensure intercoder reliability, 2 coders evaluated a common 5% (26/500) of the study sample, which is considered adequate in cases of a large sample [43]. A Cohen’s kappa coefficient of .6 was calculated, which is categorized as good agreement and is an acceptable level of intercoder reliability [44].

All analyses were conducted using Stata version 12 (StataCorp). Descriptive statistics were computed and summarized in aggregate. A total theory score was calculated by summing the values for each of the 9 construct codes (range 0-9). Combining multiple constructs to form a total theory score has been done previously [45-47] and stems from the notion that greater total amounts of theory may be most effective at changing complex diet-related behaviors [39]. Total theory scores were not normally distributed and a square root transformation was used to normalize this variable. Adjusted regression analysis was used to identify factors associated with the inclusion of HBT in pins.

## Results

### Sample Characteristics

A total of 238 infographic pins from Pinterest were analyzed. Characteristics of the pins are described in Table 1. Of these pins, 98.7% of the infographics were affiliated with a business or individual; the remainder were affiliated with a government organization. Healthy eating infographics related to either disease management or portion control constituted 32.6% (77/236) of the pins. The majority of pins were focused on very specific nutrition topics, such as lists of superfoods, health foods for babies, and nutritional content of specific fruits and vegetables. Only 33.8% (80/237) of the infographics depicted a person, and in 53.4% (126/236) of the infographics, food was depicted using cartoon figures. Under half (100/229, 43.7%) of the infographics cited references, and 34.8% (82/236) of the infographics received a recommendation by nutrition professionals. Other descriptive information is presented in Table 1. Engagement metrics, including the average number of comments, repins, and likes, are presented in Table 2.

**Table 1:** Pin characteristics.

Variables		n^a^ (%)
**Pin affiliation^b^**		
	Business	198 (83.2)
	Individual	3 (15.6)
	Government	37 (1.3)
**Pin category**	
	Macronutrients^c^	25 (10.6)
	Micronutrients^d^	36 (15.3)
	Disease management^e^	30 (12.7)
	Portion control/weight management^f^	47 (19.9)
	Other^g^	98 (41.5)
**Text^h^**		
	Text heavy (>50%)	109 (46.2)
	Text light (<50%)	127 (53.8)
**Color^i^**		
	Vibrant	155 (65.7)
	Muted	78 (33.1)
	Black and white	1 (0.4)
**Person depicted**		
	No person depicted	157 (66.2)
	Cartoon	66 (27.9)
	Photograph	14 (5.9)
**Food Depicted**		
	No food depicted	15 (6.4)
	Cartoon	126 (53.4)
	Photograph	95 (40.3)
References^j^		100 (43.7)
Professional recommendation^k^		82 (34.8)

^a^Not all categories in every variable will sum to 238 due to some instances of missing data.

^b^Pin affiliation: who authored the pin.

^c^Macronutrients: carbohydrates, proteins, fats.

^d^Micronutrients: vitamins and minerals.

^e^Disease management: cancer, obesity, arthritis, cardiovascular disease, etc.

^f^Portion control/weight management: identifying and promoting healthy portion sizes.

^g^Other: included highly specific nutrition topics including top 10 healthiest foods, lists of superfoods, harms of specific foods, and nutritional content of specific fruits and vegetables.

^h^Text: pins were categorized as text heavy if more than 50% of the infographic contained text and text light if less than 50% of the infographic contained text.

^i^Color: pins were categorized as vibrant colored, muted in color, or black and white.

^j^References: presence of references or citations.

^k^Professional recommendation: whether or not the pin provided accurate information and advice to recommend to clients.

**Table 2:** Engagement metrics.^a^

Engagement metrics	Average	SD	Range
Comments	2.35	4.37	0-30
Repins	1318	2267	3-15,848
Likes	215	440	0-5027

^a^Variables were not normally distributed and underwent transformations for analyses. The mean and SD are presented here in raw form for interpretation.

### Presence of Specific Health Behavior Theory Constructs

The prevalence of each construct [48] is presented in Table 3. Perceived benefits were present most often (170/236, 72%), followed by behavioral capability (123/238, 51.7%) and perceived severity (79/236, 33.5%). The construct that appeared the least was self-regulation/self-control (2/237, 0.8%).

### Health Behavior Theory Scores

Table 4 illustrates a summary of HBT scores according to pin affiliation. The mean total theory score was 2.03 (SD 1.2) out of a possible 9. Pins authored by an individual had the lowest mean score at 1.67, pins from nongovernment businesses had an average score of 2.09, and pins created by government organizations had the highest average score at 2.75. The pin with the highest total theory score received an HBT score of 6.

### Engagement Metrics and Health Behavior Theory

The number of repins (*P*=.04), likes (*P*=.01), and comments (*P*=.01) were positively associated with the inclusion of HBT. Including HBT constructs in an infographic was associated with greater user interaction through repins, likes, and comments (Table 5).

**Table 3:** Prevalence of health behavior theory constructs among pins.

Construct	Description [48]	n (%)
Perceived benefits	Belief about the potential positive aspects of a health action	170 (72.0)
Perceived barriers	Belief about the potential negative aspects of a health action	37 (15.6)
Perceived susceptibility	Belief about getting a disease or condition	38 (16.1)
Perceived severity	Belief about the seriousness of a condition or the consequences of leaving it unaddressed	79 (33.5)
Self-efficacy	Belief that one can achieve the behavior required to execute the outcome	11 (4.7)
Self-regulation/control	Controlling oneself through self-monitoring, goal-setting, feedback, self-reward, self-instruction, and enlistment of social support	2 (0.8)
Behavioral capability	Providing tools, resources, or environmental changes that make new behaviors easier to perform	123 (51.7)
Observational learning/modeling	Beliefs based on observing similar individuals or role models perform a new behavior	3 (1.3)
Subjective norm	An individual's perception of social norms or his/her peers' beliefs about a behavior. A function of an individual's normative beliefs and motivation to comply with beliefs	17 (7.2)

**Table 4:** Summary of health behavior theory scores.^a^

	Mean	SD	Frequency
Overall	2.03	1.20	238
Business	2.09	1.21	189
Government	2.67	0.577	3
Individual	1.67	1.12	36

^a^Variables were not normally distributed and underwent transformations for analyses. The mean and SD are presented here in raw form for interpretation.

**Table 5:** Correlation between pin engagement metrics and health behavior theory score.

	Total theory		Likes		Repins	
	Coefficient	*P* value	Coefficient	*P* value	Coefficient	*P* value
Repins	0.13	.04	0.95	<.001	1.00	—
Likes	0.16	.01	1.00	—	0.95	<.001
Comments	0.17	.01	0.66	<.001	0.64	<.001

### Pin Characteristic and Health Behavior Theory

Shown in Table 6, several characteristics were positively associated with a higher HBT score in adjusted regression analyses after controlling for other factors. Compared to a small amount of text, a large amount of text (*P*=.01) was associated with higher HBT score, as were photographs of real people (*P*=.001) (compared to no photographs of real people), cartoon pictures of food (*P*=.01) (compared to photographs of real food), and the presence of references (*P*=.001) (compared to no references). Lastly, when compared to infographics that were not coded as recommendable for professional use, infographics that received this professional recommendation had higher HBT scores (*P*=.001).

**Table 6:** Regression analysis of pin characteristic and presence of health behavior theory.

Theory square root	Coefficient	SE	*t*	*P* value	95% CI
Professional recommendation	.197	.0577	3.4	.001	.0827 to .3104
Text heavy	.141	.0547	2.58	.011	.0334 to .2491
Color	−.110	.0578	−1.89	.060	−.2237 to .0044
Photograph of person	.196	.0581	3.38	.001	.0816 to .3107
Cartoon of food	.132	.0551	2.40	.017	.0235 to .2407
References	.184	.0567	3.25	.001	.0727 to .2964

## Discussion

### Principal Findings

The purpose of this study was to determine the level of integration of HBT in nutrition infographics on Pinterest. Secondarily, we identified factors associated with its inclusion. The secondary interest was largely exploratory and was intended to inform future research and efforts that might use Pinterest to promote healthy eating. Overall, it was found that HBT constructs are integrated into nutrition pins only minimally. The low levels of HBT are not surprising, as pins can be created and shared by lay parties who may not have training in HBT or know why its inclusion may improve pin impact on behavior. This may be a recurring problem for health professionals wishing to adopt and use social media and other health technology in their health promotion efforts. Payne et al [40] and Cowan et al [45] reported similarly low levels of HBT in their respective content analyses of physical activity mobile apps and recommended increased collaboration between experts in HBT and app developers. Likewise, it may be beneficial for health professionals to partner with infographic developers to create pins. There is a rich literature base supporting the connection between HBT and healthy dietary practices [33-39]. Future research could test the impact on behavior provided that practitioners and developers were to work collaboratively to accomplish a more complete integration of HBT.

Interestingly, there were very few pins created by government organizations such as Let’s Move! and the US Department of Agriculture, although they had the highest average HBT scores. It seems plausible that pins created by government health organizations would contain higher levels of HBT because individuals working in these settings may be more likely to have training in a related discipline. In general, however, these results may indicate that businesses and individuals who create nutrition infographics for Pinterest lack the training to effectively incorporate HBT into social media campaigns. While research elsewhere indicates that health professionals currently use and understand social media in vocational roles only minimally [47,49,50], the creation of accurate nutrition infographics that include HBT by health professionals may allow Pinterest to be used as an effective health promotion tool.

The majority of pins were focused on very specific nutrition topics, such as lists of superfoods, health foods for babies, and nutritional content of specific fruits and vegetables. The second most common infographic category was portion control and weight management. It is promising that the latter category was a relatively large percentage of the sample, especially considering that portion sizes have dramatically increased over the past several decades [51] and this trend is a major contributor to the global obesity epidemic [52]. However, the high percentage of very specific nutrition topics (eg, the nutritional content of a banana or a list of healthy foods for babies) not necessarily related to preventive health behaviors such as portion control may be problematic, as providing highly specific nutrition information may not be an effective way to change health outcomes.

While Pinterest is dominated by young middle- to upper-middle-class white females, Pinterest use grew significantly among individuals living in rural locations, those with an annual salary of less than $30,000 per year, and those aged 50 years and older from 2013 to 2014 [53]. These populations are at significant risk for poor dietary behaviors and obesity in the United States [54,55], and it is not clear to what extent current infographics appeal to these populations. The lack of references incorporated in this sample is also troubling, especially as infographics containing references were more likely to contain HBT. It may also be that pins without citations are less likely to be evidence-based, an important attribute in public health interventions [56]. Health organizations creating infographics for Pinterest may want to consider tailoring pins for high-risk populations and incorporating information from reliable references to improve the accuracy of nutrition information. Including references at the bottom of infographics in a smaller font can improve credibility without hindering visual appeal.

The most common HBT constructs in this sample were perceived benefits, behavioral capability, and perceived severity. The remaining 6 constructs appeared in 15% or less of the sample, with self-regulation and control being the least common. It is concerning that self-efficacy was one of the least incorporated constructs because self-efficacy has been shown to be a significant predictor of behavior change [48] and is positively associated with better chronic disease management [29,57]. It is unsurprising that the most common HBT constructs were related to disseminating knowledge and general information; pins are largely noninteractive, especially compared to other health technology tools (apps, videos, etc), and it may be difficult to incorporate more complex HBT constructs. Public health researchers debate whether education-heavy health interventions are effective at changing behavior [58]. However, some researchers indicate that there is an association between increased nutrition knowledge and dietary behaviors, including eating smaller portions, eating foods with fewer calories, and using nutritional labels more effectively [59-61]. Additionally, given that 84% of those who access Pinterest daily report being inspired to try something new once a week or more, health information distributed through Pinterest may have the potential to influence behavior [17]. As theory-based interventions are reported to be more successful at changing behavior than those that do not contain HBT [30], Pinterest infographics containing HBT may prove to be an effective health promotion tool, although further research on the impact of infographics to change health behaviors is needed.

Factors positively associated with HBT included the presence of heavy amounts of text, a photograph of a real person, or a cartoon image of food. This suggests that depicting HBT constructs on Pinterest is more likely to be accomplished by incorporating a combination of text, people, and images rather than text alone. Indeed, research has demonstrated that images, rather than text, are the most desirable way to communicate information [62]. Less than half of nutrition pins analyzed depicted a person and even fewer included a photograph of a real person. This is not ideal, considering that realistic pictures that resonate with users are more effective at disseminating health information [29]. Including more realistic images of people engaging in healthy eating behaviors in infographics may be more likely to promote behavior change. A study with this focus might be warranted to test the impact on behavior. These findings might also guide a practitioner’s selection of images for social distribution if they perceive that such pins are more likely to include HBT.

Social media engagement is a key performance indicator that links social media usage to action [63] and can be categorized as low, medium, or high engagement. In the context of Pinterest, the number of “likes” on each pin can be considered low engagement (ie, users acknowledge or agree with content), while repins and comments can be considered medium engagement (ie, users create or share content) [29]. High engagement refers to actual offline participation and is not measurable by Pinterest engagement metrics. Although engagement with nutrition pins in the form of comments was relatively minimal in this study, findings suggest that the commenting on, repinning, and liking of nutrition-related infographics is more likely when HBT constructs are depicted. Future research could test the impact of including HBT in pins on the likelihood of offline action.

### Limitations

The researchers only assessed the image and did not evaluate the websites the pin linked to. Additionally, there are many duplicates of each pin shared on Pinterest, so engagement metrics may be spread among pins. While the researchers made note of information accuracy, it was not the primary focus of the study. A more rigorous analysis of content accuracy could be done to determine accuracy of messages.

### Conclusions

Promoting dietary modifications is an important public health strategy for preventing chronic disease. Pinterest as a social media platform has the potential to communicate health information and influence healthy eating behavior through infographics. However, current Pinterest infographics targeting healthy eating contain few HBT elements.

It is recommended that health professionals and organizations create and disseminate infographics that contain more elements of HBT to better influence healthy eating behavior and be more effective in changing behavior [30-32]. Pins should be tailored for high-risk populations and incorporate information from reliable references. This may be accomplished by including a combination of images and texts and portraying HBT constructs. In so doing, individuals (or the public) may obtain the information and skills necessary to eat healthy and prevent chronic disease. Including HBT in pins could foster increased user engagement and result in a greater likelihood of offline action.

This study may also help dietitians, public health workers, and health educators make an informed decision about whether or not to recommend Pinterest as a health information source. Because many health professionals use infographics to disseminate health information [18,19], they should take into account the accuracy of infographics on Pinterest and have a realistic expectation about whether or not pins are effective in producing behavior change.

## Abbreviations

COPD: chronic obstructive pulmonary disease
HBT: health behavior theory
